# Effect of a DIVA vaccine with and without in-feed use of coated calcium-butyrate on transmission of *Salmonella* Typhimurium in pigs

**DOI:** 10.1186/1746-6148-9-243

**Published:** 2013-12-04

**Authors:** Lotte De Ridder, Dominiek Maes, Jeroen Dewulf, Frank Pasmans, Filip Boyen, Freddy Haesebrouck, Estelle Méroc, Stefan Roels, Bregje Leyman, Patrick Butaye, Yves Van der Stede

**Affiliations:** 1 Unit of Co-ordination in Veterinary Diagnostics-Epidemiology and Risk Analysis, CODA-CERVA, Groeselenberg 99, 1180 Ukkel, Belgium; 2 Unit of Surveillance, Orientation and Veterinary Support, CODA-CERVA, Groeselenberg 99, 1180 Ukkel, Belgium; 3 Unit of Bacteriology, CODA-CERVA, Groeselenberg 99, 1180 Ukkel, Belgium; 4 Department of Obstetrics, Reproduction and Herd health, Faculty of Veterinary Medicine, Ghent University, Salisburylaan 133, 9820 Merelbeke, Belgium; 5 Department of Pathology, Bacteriology and Avian diseases, Faculty of Veterinary Medicine, Ghent university, Salisburylaan 133, 9820 Merelbeke, Belgium; 6 Department of Virology, parasitology and immunology, Faculty of Veterinary Medicine, Ghent University, Salisburylaan 133, 9820 Merelbeke, Belgium

**Keywords:** *Salmonella* Typhimurium, Pig, DIVA vaccine, Coated calcium-butyrate salt, Transmission

## Abstract

**Background:**

For satisfactory *Salmonella* control, good biosecurity along the pork production chain is crucial, although additional control measures on-farm need to be considered. This study evaluated the effect of two potential control measures against the spread of *Salmonella* Typhimurium via a transmission experiment with 56 piglets (3–15 weeks of age): two groups were orally vaccinated with 10^7^ - 10^8^ Colony Forming Units (CFU)/2 mL of a new attenuated *Salmonella* Typhimurium vaccine ‘Salmoporc-∆*rfaJ*’ with DIVA capacities (Differentiation between Infected and Vaccinated Animals) (*n* = 2x16); the feed of one group was additionally supplemented with coated calcium-butyrate salt. Two weeks post vaccination, four pigs per group were orally challenged with 10^7^ CFU/2 mL of a *Salmonella* Typhimurium strain 112910a. Both groups were compared with a positive (challenged/untreated; *n* = 16) and negative (unchallenged/untreated; *n* = 8) control group. Until six weeks post challenge, blood, individual faecal and finally tissue samples were examined. Adjusted transmission ratios ‘R_a_’ were estimated, based on the challenge strain isolation from faecal and/or tissue samples.

**Results:**

In both intervention groups, R_a_ values were lower compared to the positive control group, although these differences were not significant. In the combination group DIVA vaccine + coated butyrate, less non-challenged contact animals excreted *Salmonella* and less tissue samples were found *Salmonella*-positive in all pigs, when compared to the positive control group (*P* < 0.01). Seroconversion was detected in none of the vaccinated animals before challenge, when using a commercial lipopolysaccharide (LPS) ELISA targeting only *Salmonella* O-antigens, deleted in this vaccine. This was in contrast with an in-house whole-cell ELISA testing for various *Salmonella* antigens, in which *Salmonella*-specific antibodies were found pre-challenge in the serum of the vaccinated pigs.

**Conclusions:**

Both interventions showed a limited, non-significant reduction of *Salmonella* transmission between piglets. They may have applications towards *Salmonella* control and surveillance. Firstly, the number of *Salmonella* excreting contact pigs was significantly lower in the group where vaccination was combined with coated calcium-butyrate salt in the feed; secondly, the new vaccine confirmed its DIVA capacity. Therefore, these interventions merit further research with larger sample sizes, to optimize their use for *Salmonella* programmes.

## Background

*Salmonella* infections are one of the most important and widely distributed foodborne diseases in the European Union. Contaminated pork has been linked to 34.5% of the human outbreaks of *Salmonella enterica* subspecies *enterica* serovar Typhimurium (*Salmonella* Typhimurium) in the EU [1]. Therefore, any reduction of the *Salmonella* risk by pork products would contribute to the protection of human health. However, the control of *Salmonella* in pork production remains a significant challenge in the preharvest sector. Implementing good biosecurity and operating a high standard of hygiene is crucial in every link of the pork production chain [2], but additional control measures on-farm are indispensable [3].

Vaccination is one possible supplementary measure that can be implemented in *Salmonella* control programmes. It is currently used successfully in the poultry industry of several European countries [4,5]. Also in pigs, various vaccine studies have demonstrated a significant decrease in clinical signs and excretion of *Salmonella*[2,6-8]. Most European serosurveillance programmes however, rely on commercial lipopolysaccharide (LPS) ELISAs, which do not allow differentiation between *Salmonella*-specific antibodies induced by vaccination or by infection [9]. Several DIVA vaccines (Differentiation of Infected and Vaccinated Animals) have recently been developed [10,11], of which one vaccine, a *Salmonella* Typhimurium strain without the ‘*rfaJ*’ gene, induces antibodies undetectable with LPS ELISA [11]. Another control measure with promising features against *Salmonella* infections, is the oral administration of butyrate, especially in its coated form [12,13]. Not only does this organic acid enhance pig performance by improving gut function, it also down-regulates *Salmonella* virulence gene expression [14]. None of these studies however, have investigated the effect on the actual transmission rate of *Salmonella* between pigs.

The aim of the present study was to evaluate the effect of a commercial vaccine that was modified to a DIVA vaccine strain, and its combination with coated calcium-butyrate salt in the feed on the transmission of *Salmonella* Typhimurium in weaned pigs.

## Methods

### Study design

At three weeks of age, 56 *Salmonella*-negative piglets were randomly assigned to four different stables in the experimental animal facilities of CODA-CERVA, with two separated pens each (Table 1). Group A (*n* = 2 × 8) was orally vaccinated at four and seven weeks of age with 10^7^ - 10^8^ Colony Forming Units (CFU)/2 mL of a live LPS-mutant *Salmonella* Typhimurium strain, named the ‘Salmoporc-∆*rfaJ*’ strain, in reference to the deletion of the ‘*rfaJ*’ gene [11] in a commercial vaccine (Salmoporc®, IDT Biologika); group B (*n* = 2 × 8) was vaccinated similarly and received additionally feed supplemented with 0.3% coated calcium-butyrate salt throughout the experiment (Globamax Performant, Sanluc International); a positive control group C (challenged/untreated; *n* = 2 × 8) and a negative control group D (unchallenged/untreated; *n* = 8) were included as well. Each group, except the negative control group, consisted of two replicates to increase the power of the experiment.

**Table 1:** **Design of the transmission study with the samples**/**actions and diagnostics**/**products**, **as a function of the pigs**’ **age**

**Age of the pigs in weeks**	**Sampling or action (Group)**	**Frequency (Number of pigs)**	**Diagnostic method or product**
3 - 8	Floor faeces (group A, B, C, D)	Once/week (1 pool/pen; *n* = 7)	Isolation (ISO 6579 Annex D)
9 - 15	Rectal faeces (group A, B, C, D)	Twice/week (individually; *n* = 56)	Isolation (ISO 6579 Annex D)
3 - 15	Blood (group A, B, C, D)	Once/week (*n* = 56)	ELISA ^a^
3 - 15	Feed supplementation (group B)	*Ad libitum* (*n* = 15)	Coated calcium-butyrate salt (Globamax Performant,^,^ Sanluc International)
4 + 7	Oral vaccination (group A, B)	Twice: primer + boost	10^7^ - 10^8^ CFU of DIVA vaccine
		(*n* = 16(A); *n* = 15(B))	‘Salmoporc-∆*rfaJ*’
9	Oral challenge (group A, B, C)	Once; in a separate stable, with replacement 24 h later (2 pigs/pen; *n* = 12)	10^7^ CFU of nalidixic acid-resistant *Salmonella* Typhimurium strain 112910a
15	Rectal faeces, Ileum + content, Caecum + content, Ileocaecal Lnn, Tonsils (group A, B, C, D)	At necropsy (*n* = 56)	Isolation (ISO 6579 Annex D)

^a^Two ELISA tests were performed: (1) commercial HerdChek Swine *Salmonella* (IDEXX); (2) In-house whole-cell ELISA (Leyman *et al.*, 2011).

The pens were separated with solid concrete partitions and had a semi-solid concrete floor (half grid, half rubber mats). The pig stocking density was 0.42 m^2^ per animal. In all groups, the same starter and pig meal without antimicrobials was administered throughout the study, (*i.e.* from arrival in the experimental facilities at three weeks of age until euthanasia at 15 weeks of age), except in group B for which the feed was supplemented as described above. The pigs were housed at natural day-night rhythm with *ad libitum* access to water and feed. Each group of eight pigs was provided with a rubber ball as environmental enrichment.

With the exception of the negative control group, two pigs per pen were orally challenged at nine weeks of age with 10^7^ CFU/2 mL of a nalidixic acid-resistant *Salmonella* Typhimurium strain 112910a [15] in a separate pen. Twenty-four hours later, these challenged ‘seeder’ pigs were replaced with the naive, non-challenged contact pigs (0 Days Post Contact or ‘DPC’), and all pigs were monitored until 15 weeks of age (42 DPC). This day (0 DPC) was considered as the start of the transmission experiment. Before challenge at nine weeks of age, individual blood samples and pooled faecal pen samples were taken once a week. After challenge, blood samples were obtained once a week, whereas individual rectal faeces were collected twice a week from all pigs. At necropsy, rectal faeces, ileum, ileal content, caecum, caecal content, ileocaecal lymph nodes and tonsils were sampled. The experimental design was approved by the Animal Care and Ethical Committee of the IPH-VAR (Approval number 100412–02).

### Sample analysis

All faecal samples were examined using the ISO 6579 Annex D method [16]. Firstly, the samples were diluted 1:10 in buffered peptone water (BPW, Bio-Rad) and aerobically incubated for 16-20 h at 37°C. Of this solution, 0.1 mL was inoculated on a modified semi-solid Rappaport-Vassiliadis plate (MSRV, Bio-Rad) (one sample per plate) and aerobically incubated for 46-50 h at 41°C. After this period, a loopful of the growth area in this MSRV enrichment must be plated out on a xylose lysine deoxycholate agar plate (XLD, Bio-Rad) and another agar for choice. In this study was opted for a brilliant green agar plate (BGA, Lab M), known to allow satisfactory growth of both the vaccine and challenge strain. The latter plate was supplemented with 20 μg/mL nalidixic acid for differentiating these *Salmonella* Typhimurium strains. For 21-27 h, both plates were given an aerobic incubation at 37°C. A *Salmonella*-suspected colony on the BGA plates was expected to be only the nalidixic acid-resistant challenge strain, whereas on the XLD plates, colonies of both the challenge and vaccine strain were able to grow. From these XLD or BGA plates, one *Salmonella*-suspected colony was inoculated in triple sugar iron agar (TSI, Bio-Rad) and lysine decarboxylase bouillon (Oxoid) and incubated for 18-24 h at 37°C for final confirmation. After preparation, the tissue samples were investigated similarly to the faecal samples.

The blood samples were allowed to coagulate at room temperature and were then centrifuged for 15 min at 1200 *g*. The serum collected thereafter, was diluted twenty-fold and analysed with two ELISA tests: (1) a commercial ELISA kit based on LPS O-antigens of serogroup B, C1 and D (HerdChek Swine *Salmonella*, IDEXX); (2) an in-house whole-cell ELISA, based on a variety of surface-antigens on the *Salmonella* Typhimurium strain 112910a [11]. Consequently, antibodies against the ‘Salmoporc-∆*rfaJ*’ vaccine strain, which expresses no O-antigens, should only be detectable with this whole-cell ELISA. Other *Salmonella* strains on the other hand, will be detected in both ELISA tests. Optical densities (OD) in both ELISAs were determined by photo spectrometry with a 650 nm filter. Samples with a Sample-to-Positive (S/P) ratio ≥0.25 (= OD% ≥10) were defined as positive.

### Statistical analysis

Based upon all faecal samples, the numbers of excreting pigs were compared via generalized estimating equations (GEE), using the proc genmod procedure in SAS 9.2 (SAS Institute Inc., USA). For this, a binomial distribution and logit link function were used. Bacteriological results from the tissue samples (ileum, ileal content, caecum, caecal content, ileocaecal lymph nodes, tonsils) were compared between groups (DIVA vaccine, DIVA vaccine + Coated Butyrate, Positive Control) using logistic regression analysis in SAS 9.2. *P*-values below 0.05 were considered significant.

The transmission of *Salmonella* Typhimurium in each group was estimated on the basis of the stochastic ‘SI’ infection model (Susceptible-Infectious), using an ‘adjusted’ transmission ratio ‘R_a_’ for the observed period of six weeks, which is derived from the basic reproduction ratio R_0_ for the entire infectious period [17]. In this study, each intervention group contained *n* = 8 piglets, of which initially two challenged animals were infectious (I_0_ = 2) and six contact animals were susceptible (S_0_ = 6). An R_a_ value below or above one means respectively, that each infected animal will pass the infection on to less or more than one naive contact animal during the observed period of six weeks. This R_a_ value was estimated via the Maximum Likelihood Estimation (MLE) based on the final size of the study, which represents the total number of contact infections [18]. A contact animal was considered infected, when at least one sample was positive in: i) individual faeces, or ii) ileum, caecum and/or their content, or iii) ileocaecal lymph nodes and/or tonsils, or iv) all tissues and/or all faeces collected. R_a_ values were calculated for each of the previous four categories, and significant differences between groups were assessed [19].

## Results

### Bacteriological examination

In each group, all pooled faecal samples taken prior to challenge were *Salmonella*-negative. All individual faecal samples from the negative control group remained negative during the whole experiment. One contact pig of group B died at -1 DPC due to a polybacterial bronchopneumonia and was excluded from the study (no respiratory pathogens could be identified at necropsy).

**Figure 1:**
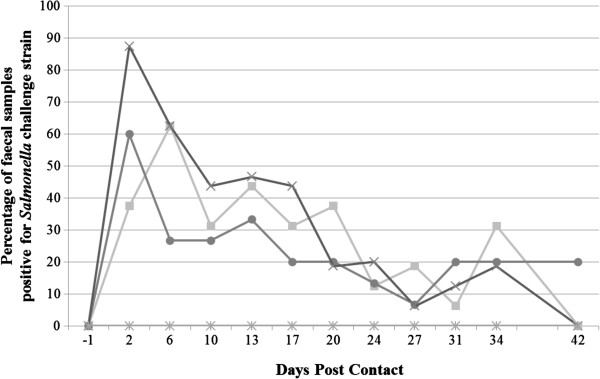
**Proportion of *Salmonella *challenge strain positive faecal samples per groups, as a function of time (DPC: days post contact).** (■) DIVA vaccine; (●) DIVA vaccine + Coated calcium-butyrate salt; (✖) Positive Control; (✱) Negative Control. DPC = -1: Replacement and oral challenge with 10^7^ CFU/2 mL *Salmonella* Typhimurium of 2 seeder pigs per group of 8 pigs, with reintroduction of seeders in their original pen 24 h later.

After challenge, no significant differences between the groups were observed in the numbers of *Salmonella*-excreting animals, when considering all challenged and non-challenged pigs (Figure 1). If the challenged seeder pigs were not taken into account for all 11 sampling points however, significantly fewer *Salmonella*-positive samples were obtained in group B (9/121), when compared to group C (30/132) (*P* < 0.01). At the end of the trial, respectively 8/12 (67%), 7/11 (64%) and 12/12 (100%) non-challenged contact pigs from group A, B and C had excreted *Salmonella* in their feces on at least one sampling occasion (Additional file 1: Table S1). From all necropsy samples of both challenged and non-challenged pigs, 27, 13 and 41% were found positive for the challenge strain in group A, B and C, respectively, with a significant difference between group B and C (*P* < 0.01) (Table 2). Respectively 6/12 (50%), 2/11 (18%) and 10/12 (83%) non-challenged contact pigs from group A, B and C had one tissue sample or more colonized with *Salmonella*. Both the faecal shedding and tissue colonization are presented individually in Additional file 1: Table S1.

**Table 2:** **Results of the descriptive and logistic regression analysis of the necropsy samples positive for the *Salmonella *** **challenge strain in the three groups**

**Group **^**§**^	**Parameters**	**Different necropsy samples**						**All necropsy samples**
		**Ileum**	**Ileal Content**	**Caecum**	**Caecal Content**	**Ileocaecal Lnn**	**Tonsils**	
A) DIVA vaccine (*n* = 16)	Number	4 ^a^	4 ^ab^	6 ^a^	5 ^ab^	3 ^a^	4 ^a^	26 ^ab^
	(Proportion)	(25%)	(25%)	(38%)	(31%)	(19%)	(25%)	(27%)
	OR*	0.67	0.21	2.23	0.23	0.49	0.25	0.55
	*P*-value	0.66	0.10	0.38	0.07	0.41	0.18	0.05
B) DIVA vaccine + Coated Butyrate (*n* = 15)	Number	2 ^a^	1 ^a^	2 ^a^	3 ^a^	1 ^a^	3 ^a^	12 ^a^
	(Proportion)	(13%)	(7%)	(13%)	(20%)	(7%)	(20%)	(13%)
	OR*	0.25	0.03	0.34	0.12	0.15	0.14	0.30
	*P*-value	0.18	0.01	0.33	0.02	0.10	0.08	<0.01
C) Positive control (*n* = 16) (ref)	Number	5 ^a^	8 ^b^	4 ^a^	10 ^b^	5 ^a^	7 ^a^	39 ^b^
	(Proportion)	(31%)	(50%)	(25%)	(63%)	(31%)	(44%)	(41%)

^a, b^Numbers in a column, not sharing the same letters in superscript, are significantly different: ileal and caecal content (*P* < 0.02) and all necropsy samples (*P* < 0.01).

*Odds Ratios (*OR*) >1 or <1, define the intervention of group A or B as a risk or protective factor, respectively, for having *Salmonella*-positive tissue samples, when compared to the positive control group.

^§^A) oral vaccination at four and seven weeks of age with 10^7^ - 10^8^ CFU/2 mL of the live ‘Salmoporc-∆*rfaJ*’ strain; B) vaccination of (A) plus feed supplemented with 0.3% coated calcium-butyrate salt; C) positive control that was challenged without being treated.

The adjusted transmission ratios R_a_, based on the faecal and/or tissue results of all challenged and non-challenged pigs, were lower in all four categories in both vaccinated groups A and B, when compared to the unvaccinated group C (Table 3). These differences between groups were not significant (*P* > 0.05).

**Table 3:** **Adjusted reproduction ratio R**_**a**_ [**95**% **confidence interval**] **for groups A**, **B**, **C**, **as a function of four different categories**

**Group **^**§**^	**Individual faeces **^**a**^	**Ileum/content/Caecum/content **^**a**^	**Ileocaecal Lnn/tonsils **^**a**^	**All tissues/individual faeces **^**a**^
A) DIVA vaccine (*n* = 16)	1.76 [1.02; 9.01]	1.03 [0.56; 3.95]	0.77 [0.35; 3.80]	2.37 [1.46; 10.02]
B) DIVA vaccine + Coated Butyrate (*n* = 15)	2.52 [0.99; 9.62]	0.50 [0.17; 5.82]	0.37 [0.14; 4.64]	2.52 [0.99; 9.62]
C) Positive control (*n* = 16) *	+∞ [1.88; +∞]	2.52 [1.02; 9.01]	1.19 [0.70; 7.18]	+∞ [1.88; +∞]
*P*-value (A, C)	0.22	0.25	0.21	0.37
*P*-value (A, B)	0.47	0.38	0.21	0.27
*P*-value (B, C)	0.19	0.31	0.07	0.19

^a^Using the *ISO* method 6579 Annex D, a pig was considered as infected, if at least one of the samples of four marked categories in every column was found positive during the transmission period.

^§^A) oral vaccination at four and seven weeks of age with 10^7^ - 10^8^ *CFU*/2 mL of the live ‘Salmoporc-∆*rfaJ*’strain; B) vaccination of (A) plus feed supplemented with 0.3% coated calcium-butyrate salt; C) positive control that was challenged without being treated.

*+∞ = plus infinity.

### Serological examination

**Figure 2:**
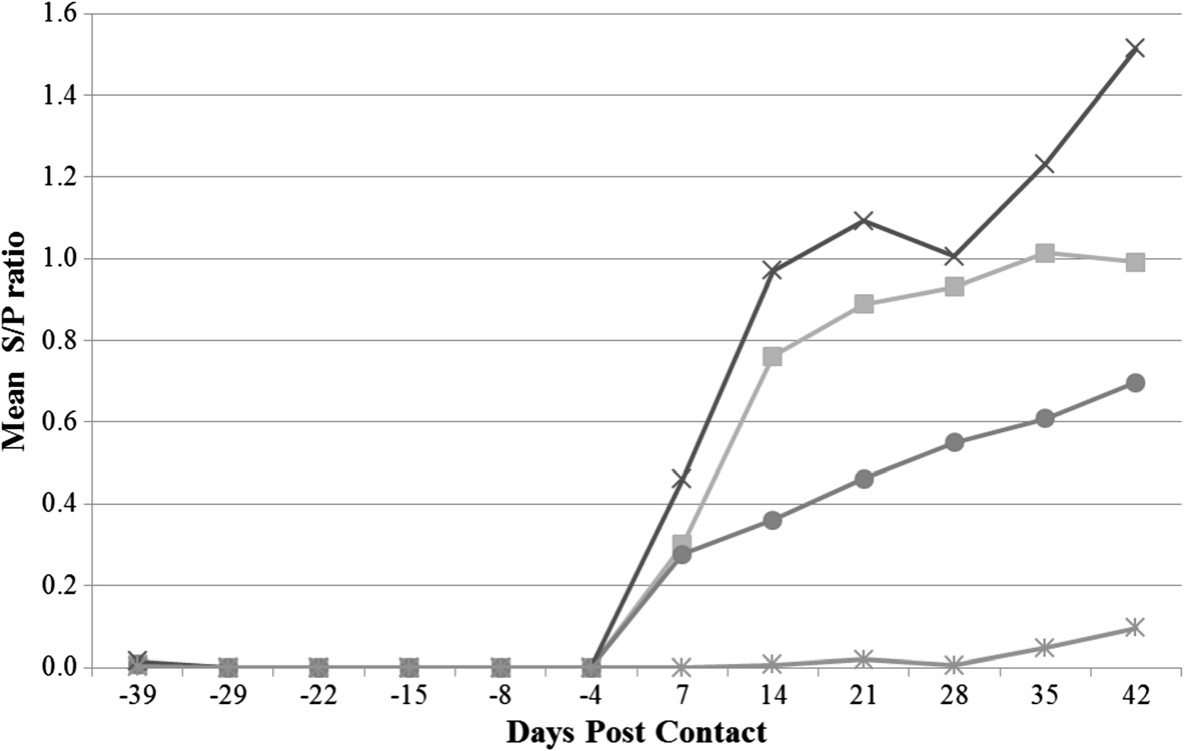
**Weekly detection** (**LPS ELISA**, **IDEXX**) **of *Salmonella***-**specific antibodies (mean S/P ratio), as a function of time (DPC: days post contact).** (■) DIVA vaccine; (●) DIVA vaccine + Coated calcium-butyrate salt; (✖)Positive Control; (✱) Negative Control. DPC = -1: Replacement and oral challenge with 10^7^ CFU/2 mL *Salmonella* Typhimurium of 2 seeder pigs per group of 8 pigs, with reintroduction of seeders in their original pen 24 h later.

In the commercial LPS-ELISA, all pigs were seronegative for *Salmonella*-specific antibodies before challenge (Figure 2). In contrast, the in-house whole-cell ELISA showed that the mean S/P ratio increased already one week before challenge in both vaccinated groups A and B, whereas the increase occurred two weeks after challenge in the positive control group C (results not shown).

## Discussion

The increase of global meat consumption during the next decades is expected to be largely due to increases in pork [20]. The reduction of any pig-related public health risk is therefore of considerable importance. *Salmonella* Typhimurium represents a most relevant pig-associated risk despite current control measures [1], and was therefore selected for the present transmission experiment in weaned pigs. This study aimed to assess the effect of a DIVA vaccine [11], with and without coated calcium-butyrate salt in the feed.

As a consequence of the study design (transmission experiment), in which the treatment was applied to all pigs before challenge, the combined treatment effect on both the excretion of the inoculated pigs and the susceptibility of the contact animals is evaluated. This is an important difference with efficacy studies in which mainly the susceptibility is assessed and not the combined effect [6,21,22]. This study concept also allows to better simulate the field situation, namely infection of a treated pig which may subsequently infect other treated pigs in the area. Several other bacterial diseases have been investigated in similar experiments [18,23-30]. As the R_a_ values were higher than one in this experiment, the present interventions will not eradicate infection with *Salmonella* Typhimurium during the studied period. However, the treated groups demonstrated non-significantly lower R_a_ values for every matrix, in comparison with the positive control group. Therefore, a more distinct reducing effect might be expected when observing pigs for a longer time, as was demonstrated in previous efficacy studies [7,31,32], which investigated the efficacy of vaccination, acidified drinking water, and fermented liquid feed during the entire finishing period, respectively. This expected enlarged difference between groups might be explained by a prolonged potential spread of *Salmonella* in the untreated control group, while no further spread is expected in the treatment groups.

Concerning vaccination, different studies have demonstrated a reduction of *Salmonella* shedding and/or *Salmonella* seroprevalence in pigs [6,7,10,33,34]. Vaccine-induced antibodies should be distinguishable however, from those induced by infection in order not to hamper monitoring programmes based on serology. Selke *et al.*[10] developed a DIVA vaccine of which the induced antibodies were not detectable in their in-house ELISA, but the DIVA vaccine of Leyman *et al.*[11] induced antibodies undetectable with a commercial LPS ELISA that is commonly used in serosurveillance programmes (HerdChek Swine *Salmonella*, IDEXX). Consequently, the latter vaccine would be suitable for use in European serosurveillance programmes, whereas the first vaccine would not. In the present study, the method of Leyman *et al.*[11] was therefore applied on the commercial vaccine Salmoporc®, resulting in the ‘Salmoporc-∆*rfaJ*’ strain. The commercial vaccine Salmoporc® has proven its effectiveness previously [33] using the same administration procedure as the current study, and Leyman *et al.*[11] demonstrated a similar protection after immunization with either a wild-type *Salmonella* Typhimurium strain or its DIVA variant. Therefore, the ‘Salmoporc-∆*rfaJ*’ strain used in this study was expected to have the same protective effect as the Salmoporc® parent strain. Whereas the in vivo study of Leyman *et al.*[11] involved four unchallenged vaccinated piglets, this new DIVA strain was evaluated in 16 challenged piglets. Although the ‘Salmoporc-∆*rfaJ*’ strain as single intervention resulted in a limited non-significant reduction of transmission, serological surveillance using the commercial ELISA-tests remains applicable. Therefore, it may be considered a promising tool in future *Salmonella* surveillance, worthy of further investigation for optimization.

Previous studies with organic acids in the feed showed both reducing [22,35] and non-reducing [36,37] capacities on *Salmonella* excretion or antibody induction. In this study, we opted for supplementing the feed with coated calcium-butyrate, as earlier studies [12,13] have shown that coating of fatty acids is needed to let them reach the colonization sites (ileum, caecum and colon) in their active form. In addition, the in-feed coated butyrate was combined with the current DIVA vaccine, as these two control strategies are believed to be complementary. Namely, both strategies have a different working mechanism to combat *Salmonella* transmission, *i.e.* the vaccination enhancing the host’s immune response and the coated butyrate targeting *Salmonella* bacteria in the gut environment and improving intestinal epithelial growth. In the current study, *Salmonella* transmission in the DIVA vaccine + butyrate group was not significantly different from the one in both other challenged groups. However, in this group significantly less non-challenged contact animals excreted *Salmonella* and significantly less organ samples of all challenged and non-challenged pigs were found *Salmonella*-positive, when compared to in the positive control group. An additional beneficial effect was thus observed in the combination group, in comparison with the DIVA vaccine intervention on its own, which might be explained by this dual approach to *Salmonella* infection. However, the combination of both strategies would also incur considerable additional costs, in comparison with the single interventions. Therefore, more research including a cost-benefit analysis and while observing pigs for a longer period (*e.g.* from weaning till market age), is warranted.

## Conclusions

Both interventions in this study did not show a significant reduction of *Salmonella* Typhimurium transmission. Significantly less contact pigs excreted *Salmonella* however, in the group where vaccination was combined with coated butyrate in the feed, and the vaccine itself confirmed its ‘DIVA’ capacity. Therefore, these interventions merit further research to improve their applicability in *Salmonella* control programmes.

## Abbreviations

BGA: Brilliant green agar; BPW: Buffered peptone water; CFU: Colony forming units; DIVA: Differentiation between infected and vaccinated animals; DPC: Days post contact; ELISA: Enzyme-linked immuno sorbent assay; GEE: Generalized estimating equations; Lnn: Lymph nodes; LPS: Lipopolysaccharide; MLE: Maximum likelihood estimation; MSRV: Modified semi-solid rappaport-vassiliadis; OD: Optical density; S/P ratio: Sample-to-positive ratio; TSI: Triple sugar iron; XLD: Xylose lysine deoxycholate.

## Competing interests

The authors declare that they have no competing interests.

## Authors’ contributions

DM, JD, PB and YVdS all participated in the design and coordination of the study. All authors helped to draft the manuscript. BL provided the vaccine strain and the whole-cell ELISA. LDR carried out the herd screening, vaccination and sampling, performed the bacteriological isolation and serological assays, participated in the statistical analysis. SR, EM and YVdS helped performing the necropsy. YVdS and JD performed the statistical analysis. All authors read and approved the final manuscript.

## Supplementary Material

Additional file 1: Table S1 Presentation of the individual *Salmonella***-**positive faecal and necropsy samples **(**grey**)** per sampling occasion and tissue**,** respectively. Click here for file
