# Supplementary material for: Effect of a DIVA vaccine with and without in-feed use of coated calcium-butyrate on transmission of Salmonella Typhimurium in pigs
Source: BMC Vet Res. 2013 Dec 4;9:243. doi: 10.1186/1746-6148-9-243 (PMC4235050; doi:10.1186/1746-6148-9-243)
Supplement: Additional file 1: Table S1 — Presentation of the individual Salmonella-positive faecal and necropsy samples (grey) per sampling occasion and tissue, respectively. [file 1746-6148-9-243-S1.doc]

**Table S1:** Presentation of the individual Salmonella-positive faecal and necropsy samples (grey) per sampling occasion and tissue, respectively

| **Group** ^§^ | **Pig ^a^** | **Faeces** | | | | | | | | | | | | | **Tissue ^b^** | | | | | | |
| --- | --- | --- | --- | --- | --- | --- | --- | --- | --- | --- | --- | --- | --- | --- | --- | --- | --- | --- | --- | --- | --- |
|  |  | **Days Post Challenge** | | | | | | | | | | | | **Excreting pig** | **I** | **Ic** | **C** | **Cc** | **L** | **T** | **Colonized Pig** |
|  |  | **-1** | **2** | **6** | **10** | **13** | **17** | **20** | **24** | **27** | **31** | **34** | **42** |  |  |  |  |  |  |  |  |
| A) DIVA vaccine (*n* = 16) | *S* |  |  |  |  |  |  |  |  |  |  |  |  | *Yes* |  |  |  |  |  |  | *Yes* |
|  | *S* |  |  |  |  |  |  |  |  |  |  |  |  | *Yes* |  |  |  |  |  |  | *Yes* |
|  | C |  |  |  |  |  |  |  |  |  |  |  |  | No |  |  |  |  |  |  | No |
|  | C |  |  |  |  |  |  |  |  |  |  |  |  | No |  |  |  |  |  |  | No |
|  | C |  |  |  |  |  |  |  |  |  |  |  |  | Yes |  |  |  |  |  |  | Yes |
|  | C |  |  |  |  |  |  |  |  |  |  |  |  | No |  |  |  |  |  |  | Yes |
|  | C |  |  |  |  |  |  |  |  |  |  |  |  | Yes |  |  |  |  |  |  | No |
|  | C |  |  |  |  |  |  |  |  |  |  |  |  | Yes |  |  |  |  |  |  | Yes |
|  | *S* |  |  |  |  |  |  |  |  |  |  |  |  | *Yes* |  |  |  |  |  |  | *Yes* |
|  | *S* |  |  |  |  |  |  |  |  |  |  |  |  | *Yes* |  |  |  |  |  |  | *Yes* |
|  | C |  |  |  |  |  |  |  |  |  |  |  |  | Yes |  |  |  |  |  |  | No |
|  | C |  |  |  |  |  |  |  |  |  |  |  |  | Yes |  |  |  |  |  |  | Yes |
|  | C |  |  |  |  |  |  |  |  |  |  |  |  | Yes |  |  |  |  |  |  | No |
|  | C |  |  |  |  |  |  |  |  |  |  |  |  | Yes |  |  |  |  |  |  | Yes |
|  | C |  |  |  |  |  |  |  |  |  |  |  |  | Yes |  |  |  |  |  |  | Yes |
|  | C |  |  |  |  |  |  |  |  |  |  |  |  | No |  |  |  |  |  |  | No |
| B) DIVA vaccine + Coated Butyrate (*n* = 15) | *S* |  |  |  |  |  |  |  |  |  |  |  |  | *Yes* |  |  |  |  |  |  | *Yes* |
|  | *S* |  |  |  |  |  |  |  |  |  |  |  |  | *Yes* |  |  |  |  |  |  | *Yes* |
|  | C |  |  |  |  |  |  |  |  |  |  |  |  | Yes |  |  |  |  |  |  | Yes |
|  | C |  |  |  |  |  |  |  |  |  |  |  |  | Yes |  |  |  |  |  |  | No |
|  | C |  |  |  |  |  |  |  |  |  |  |  |  | Yes |  |  |  |  |  |  | No |
|  | C |  |  |  |  |  |  |  |  |  |  |  |  | Yes |  |  |  |  |  |  | No |
|  | C |  |  |  |  |  |  |  |  |  |  |  |  | Yes |  |  |  |  |  |  | No |
|  | C |  |  |  |  |  |  |  |  |  |  |  |  | Yes |  |  |  |  |  |  | Yes |
|  | *S* |  |  |  |  |  |  |  |  |  |  |  |  | *Yes* |  |  |  |  |  |  | *No* |
|  | *S* |  |  |  |  |  |  |  |  |  |  |  |  | *Yes* |  |  |  |  |  |  | *Yes* |
|  | C | - | - | - | - | - | - | - | - | - | - | - | - | - | - | - | - | - | - | - | - |
|  | C |  |  |  |  |  |  |  |  |  |  |  |  | No |  |  |  |  |  |  | No |
|  | C |  |  |  |  |  |  |  |  |  |  |  |  | No |  |  |  |  |  |  | No |
|  | C |  |  |  |  |  |  |  |  |  |  |  |  | No |  |  |  |  |  |  | No |
|  | C |  |  |  |  |  |  |  |  |  |  |  |  | No |  |  |  |  |  |  | No |
|  | C |  |  |  |  |  |  |  |  |  |  |  |  | Yes |  |  |  |  |  |  | No |
| C) Positive control (*n* = 16) | *S* |  |  |  |  |  |  |  |  |  |  |  |  | *Yes* |  |  |  |  |  |  | *Yes* |
|  | *S* |  |  |  |  |  |  |  |  |  |  |  |  | *Yes* |  |  |  |  |  |  | *Yes* |
|  | C |  |  |  |  |  |  |  |  |  |  |  |  | Yes |  |  |  |  |  |  | Yes |
|  | C |  |  |  |  |  |  |  |  |  |  |  |  | Yes |  |  |  |  |  |  | Yes |
|  | C |  |  |  |  |  |  |  |  |  |  |  |  | Yes |  |  |  |  |  |  | No |
|  | C |  |  |  |  |  |  |  |  |  |  |  |  | Yes |  |  |  |  |  |  | No |
|  | C |  |  |  |  |  |  |  |  |  |  |  |  | Yes |  |  |  |  |  |  | Yes |
|  | C |  |  |  |  |  |  |  |  |  |  |  |  | Yes |  |  |  |  |  |  | Yes |
|  | *S* |  |  |  |  |  |  |  |  |  |  |  |  | *Yes* |  |  |  |  |  |  | *Yes* |
|  | *S* |  |  |  |  |  |  |  |  |  |  |  |  | *Yes* |  |  |  |  |  |  | *Yes* |
|  | C |  |  |  |  |  |  |  |  |  |  |  |  | Yes |  |  |  |  |  |  | Yes |
|  | C |  |  |  |  |  |  |  |  |  |  |  |  | Yes |  |  |  |  |  |  | Yes |
|  | C |  |  |  |  |  |  |  |  |  |  |  |  | Yes |  |  |  |  |  |  | Yes |
|  | C |  |  |  |  |  |  |  |  |  |  |  |  | Yes |  |  |  |  |  |  | Yes |
|  | C |  |  |  |  |  |  |  |  |  |  |  |  | Yes |  |  |  |  |  |  | Yes |
|  | C |  |  |  |  |  |  |  |  |  |  |  |  | Yes |  |  |  |  |  |  | Yes |

^a^ S = Seeder pig (in italics); C = Contact pig.

^b^ Tissue*:* I *=* Ileum, Ic = Ileal content, C *=* Caecum, Cc *=* Caecal content, L = Ileocaecal Lymph nodes, T = Tonsils.

^§^ A) oral vaccination at four and seven weeks of age with 10^7^ - 10^8^ CFU/2 mL of the live ‘Salmoporc-∆rfaJ’-strain; B) vaccination of (A) plus feed supplemented with 0.3% coated calcium-butyrate salt; C) positive control that was challenged without being treated
